# Rapid adaptations of *Legionella pneumophila* to the human host

**DOI:** 10.1099/mgen.0.000958

**Published:** 2023-03-22

**Authors:** Daniël Leenheer, Anaísa B. Moreno, Kiran Paranjape, Susan Murray, Sophie Jarraud, Christophe Ginevra, Lionel Guy

**Affiliations:** ^1^​ Department of Medical Biochemistry and Microbiology, Science for Life Laboratory, Uppsala University, Uppsala, Sweden; ^2^​ Ph.D. Program in Human Biology, School of Integrative and Global Majors, University of Tsukuba, Tsukuba, Japan; ^3^​ French National Reference Center of Legionella, Institute of Infectious Agents, Hospices Civils de Lyon, Lyon, France; ^4^​ CIRI, Centre International de Recherche en Infectiologie, Legionella Pathogenesis Team, Inserm, U1111, Université Claude Bernard Lyon 1, CNRS, UMR5308, ENS de Lyon, Lyon, France

**Keywords:** comparative genomics, host-specific adaptations, *Legionella pneumophila*, Legionnaires’ disease, molecular evolution

## Abstract

*
Legionella pneumophila* are host-adapted bacteria that infect and reproduce primarily in amoeboid protists. Using similar infection mechanisms, they infect human macrophages, and cause Legionnaires’ disease, an atypical pneumonia, and the milder Pontiac fever. We hypothesized that, despite the similarities in infection mechanisms, the hosts are different enough that there exist high-selective value mutations that would dramatically increase the fitness of *Legionella* inside the human host. By comparing a large number of isolates from independent infections, we identified two genes, mutated in three unrelated patients, despite the short duration of the incubation period (2–14 days). One is a gene coding for an outer membrane protein (OMP) belonging to the OmpP1/FadL family. The other is a gene coding for an EAL-domain-containing protein involved in cyclic-di-GMP regulation, which in turn modulates flagellar activity. The clinical strain, carrying the mutated EAL-domain-containing homologue, grows faster in macrophages than the wild-type strain, and thus appears to be better adapted to the human host. As human-to-human transmission is very rare, fixation of these mutations into the population and spread into the environment is unlikely. Therefore, parallel evolution – here mutations in the same genes observed in independent human infections – could point to adaptations to the accidental human host. These results suggest that despite the ability of *L. pneumophila* to infect, replicate in and exit from macrophages, its human-specific adaptations are unlikely to be fixed in the population.

## Data Summary

The sequencing data generated in this study are available in the NCBI database under the BioProject accession number: PRJEB52976.

**Impact Statement**

*
Legionella pneumophila* primarily infect amoeboid protists, but occasionally also human lung macrophages, causing Legionnaires’ disease, an atypical pneumonia. By comparing 171 isolates from patients to their probable environmental source, we identified 123 mutations that presumably occurred, or were selected, in-patient. Several genes displayed two or more independent mutations. In particular, two genes were each mutated in three patient samples, significantly more often than expected by chance alone, and are likely to represent adaptations to the human host. We experimentally show that, for one mutation at least, the mutated strain grows faster in human macrophages than in amoebae. By specifically investigating mutations occurring or selected in patients, we were able to identify two genes that might be involved in human host-specific adaptations of *L. pneumophila *. This result suggests that *L. pneumophila* is not particularly well adapted to the human host, as mutations get fixed or selected in-patient, during the short course of an infection (2–14 days).

## Introduction

### Legionella

*Legionella* are Gram-negative bacteria, belonging to the *Gammaproteobacteria *, commonly found in aquatic and soil environments [1], where they are able to infect a wide variety of protozoan hosts ranging from free-living amoebae to ciliated protozoa [2, 3]. Inside these hosts, *Legionella* are able to resist killing by water disinfection procedures commonly employed in human-made potable water systems [4]. Human exposure commonly occurs via the inhalation of contaminated aerosols produced from these systems, through showers, taps, fountains, etc. In the past two decades, cases of Legionnaires’ disease have dramatically increased, with an estimated 8–10-fold surge between 2000 and 2018 [5]. The fatality rate is high, about 8%, with the elderly, males, smokers and the immunosuppressed at a higher risk of contracting the disease.

Several *Legionella* species are known to be capable of infecting mammalian cells, such as alveolar macrophages inside the human lung, with *Legionella pneumophila* being the most frequent human pathogen. In susceptible cases, *Legionella* infection or legionellosis may lead to a severe, atypical pneumonia known as Legionnaires’ disease, or the milder Pontiac fever [6, 7]. Legionnaires’ disease typically lasts 2–14 days, and ends up by *Legionella* being cleared by the immune system, or by the death of the patient [8]. Except for one documented case, human-to-human transmission appears to be very rare [9].

### Substitution rate in *L. pneumophila*

Estimates of bacterial substitution rates were initially believed to be in the order of 10^−10^ to 10^−9^ substitutions per site per year [10, 11]. However, studies have shown that short-term evolution rates are more likely to be in the order of 10^−9^ to 10^−5^ substitutions per site per year [12, 13]. With the advances in whole genome sequencing, it has become possible to sequence multiple isolates from the same host. This has led to several studies comparing pairs of genomes from within the same host, such as *Mycobacterium tuberculosis* [14], *Escherichia coli* [15], *Clostridium difficile* [16], *Staphylococcus aureus* [17], *Klebsiella pneumoniae* [18] and *Helicobacter pylori* [19], with within-host point mutation rates ranging from 0.5 to 30 mutations per genome per year. The evolutionary rate of *L. pneumophila* ST578 strains from Alcoy, Spain, is estimated at ~10^−5^ substitutions per site per year [20]. This translates to a lower bound estimate of approximately 35 SNPs per genome per year, or 0.2–1.3 SNPs per genome for the average (2–14 day) incubation period. However, these rates are for substitutions, with mutation rates (and especially high-selective value mutations) probably occurring at higher rates.

### Recombinations in *L. pneumophila*

Substitutions occur through point mutation, but also through recombination, following transduction, conjugation or transformation. The former two mechanisms have been described in *L. pneumophila* [21, 22], and comparative genomics revealed that recombination is actually responsible for most substitutions in this organism [23–25]. The majority of recombinations occur in a few hot-spots, which include regions encoding outer membrane proteins, the lipopolysaccharide (LPS) region and effectors secreted by the Type IVB Secretion System (T4BSS) [24].

### Adaptations to the human host

This study aims to identify human-specific adaptations to the human host in *L. pneumophila *, and, in a larger context, to test whether humans are an evolutionary dead-end for *L. pneumophila. L. pneumophila* live in environments where they meet many different hosts. The different hosts steer the evolution and gene content in *L. pneumophila *, in a combinatorial way, differently for different strains of *L. pneumophila* [26]. The selective effect of the human host is unlike the one in protist hosts: human-to-human transmission appears to be a very rare event – only a single case has been documented thus far [9] – and *L. pneumophila* infections either get cleared by the immune system or result in the patient’s death. Thus, human-specific adaptations resulting from *L. pneumophila* growing inside human macrophages are unlikely to be fixed and spread, either to other human hosts or to the environment, as there is no apparent way for *L. pneumophila* to get back to an environment where they would meet human macrophages again. Therefore, we hypothesize that mutations in a number of genes would provide *L. pneumophila* with a strong selective advantage in infecting and colonizing the human host. These mutations, occurring during the short incubation period of Legionnaires’ disease (2–14 days), would not be present in the environmental *L. pneumophila* population and would be happening again and again in independent cases of Legionnaires’ disease. These mutations, conferring a selective advantage in macrophages but not in amoebae, could be considered as human-specific adaptations.

In this contribution, we compared 171 pairs of strains, one from a single infection or an outbreak, and the other from its inferred environmental source. We identified several mutations occurring in the same gene in independent infections, more often than by chance. We propose that these mutations, which may have occurred in the patient or in the environment, have been selected while inside macrophages, yield a selective advantage and thus represent human-specific adaptations.

## Methods

### Bacterial samples

Clinical (grown from sputum samples) and environmental samples were shipped to Uppsala by the Public Health Agency of Sweden (48 samples), the French National Reference Center for *Legionella* (96 samples), and the Spanish Laboratorio de *Legionella* (27 samples) on buffered charcoal yeast extract (BCYE) agar plates. Bacteria were collected directly from these plates, to minimize the number of generations and the accumulation of non-relevant mutations prior to sequencing.

### DNA isolation and sequencing

DNA was extracted from 154 clinical and environmental samples using the MasterPure DNA Purification Kit (Epicentre), following the manufacturer’s protocol. The quantity and quality of extracted DNA was assessed by NanoDrop (ThermoFisher) and gel electrophoresis on a 1 % agarose gel. Extracted DNA was prepared and sequenced using MiSeq v3 (Illumina), using 300 bp paired-end sequencing at the National Genomics Infrastructure (NGI) Sweden, SciLifeLab, Stockholm, Sweden.

A literature study identified 66 published *L. pneumophila* samples for which clinical and potential environmental samples could be identified [20, 27–34] (Table S1, available with the online version of this article).

### Assembly

Read quality was controlled using FastQC [35] and MultiQC [36]. Reads were trimmed with SeqPrep [37], and *de novo* assembled using SPAdes 3.9.1 [38]. Contigs of short length (<500 bp) and low coverage (<10×) were removed and remaining contigs were annotated using prokka 1.12-beta [39].

### Variant calling

Genetic variants (SNPs and indels) in clinical samples were called with RedDog [40], using the corresponding assembled environmental samples as reference. In the few cases where there was more than one environmental sample available, the one with the fewest SNPs was chosen for reference. Samples with >20 SNPs were removed from the analysis, as in these, the time of divergence between the environmental strain and the patient strain is presumably too long and most SNPs are likely to have occurred outside of the patient. As a control all samples were subjected to self-to-self variant calling, in which variants were called with the reads for each sample against their assembly. Samples with more than ten SNPs called against themselves were removed from the analysis. The annotated genomes and RedDog variant positions were used to confirm if mutations were synonymous or non-synonymous.

### Orthologs clustering

All proteomes from the SPAdes assemblies and from five reference genomes (Alcoy, NC_014125; Corby, NC_009494.2; Philadelphia, NC_002942.5; Lens, NC_006369; Lorraine, NC_018139.1) were clustered into ortholog protein families using OrthoMCL [41]. Variants were then matched to their respective proteome to identify the consequences of the mutations, and to which protein family they belonged. Annotation of the protein families in which multiple variants were found between independent samples was done by searching for the protein accession numbers in the first reference genome and using each region as a query for a blast search.

### Simulation of non-selective environment

To estimate the distribution of mutations per gene to expect in a non-selective environment, we redistributed 123 SNPs, the same number that were observed in this study, randomly across the genes of *L. pneumophila* Paris. To account for genes that cannot be mutated, we excluded essential genes, using a conservative estimate of 588 essential genes [42], whereas other studies suggest a number of essential genes below 400 [43, 44]. The simulation was also repeated using only 1000 genes able to accept mutations. The number of genes receiving an SNP once or several times was recorded. The sampling was repeated 1000 times. The probability that the observed number of genes mutated twice and three times, respectively, would be greater than in a non-selective environment was assessed by comparing the observed results (two genes mutated three times, seven genes mutated twice, 103 genes mutated once) to the distribution obtained from the 1000 simulations, using one-sided Mann–Whitney tests, via wilcox.test in the stats package of R [45]. The results were visualized with ggplot2 [46].

### Phylogenomics

A phylogenetic tree was generated based on a core genome multilocus sequence typing (cgMLST) scheme via the ChewBBaca pipeline [47]. The already available *L. pneumophila* Philadelphia (NC_002942.5) strain training file was used. Loci present in 95 % of genomes were included. The nucleotide sequences were aligned using MAFFT-LINSI [48]. A phylogenetic tree was produced using FastTree [49] with the Jukes–Cantor substitution model.

### Functional annotation

An amino acid sequence search in the RCSB Protein Data Bank [50] was used to identify homologous structures, and protein sequences were then aligned using MAFFT-LINSI. The structural effects of the mutations were predicted via NetSurfP-2.0 [51]. Mutations were visualized on the predicted structures of *Pseudomonas aeruginosa* homologues (PDB accessions: 3DWO for the OmpP1/FadL homologue and 4RNH for the EAL-containing protein) via YASARA View [52].

### Identification of variants in databases

To assess whether the mutations identified in the two genes lpg0707 and lpg0891 were present in other sequenced strains, we assembled an *L. pneumophila *-specific protein database, using SDBW [53]. The database was queried with blastp [54], using the one representative of each of the two three-times mutated genes as query, and an E-value threshold of 10^−6^. The results were then filtered and only proteins 80 % or more similar to the query were kept. All resulting hits were retrieved from the database, and aligned with MAFFT-LINSI [48]. The variations in amino acid residues and in length were examined in Seaview [55].

### Bacterial strains, cell culture and media

*Legionella* strains used were cultured in CYE [1 % ACES, 1 % yeast extract, 0.2 % charcoal, 1.5 % agar, 0.025 % iron (III) pyrophosphate, 0.04 % l-cysteine, pH 6.9] plates or ACES yeast extract (AYE) [1 % ACES, 1 % yeast extract, 0.025 % iron (III) pyrophosphate, 0.04 % l-cysteine, pH 6.9] broth at 37°C, unless otherwise stated. The relationship between absorbance (OD_600_) and c.f.u. ml^-1^ was established for each strain studied.

*Acanthamoeba castellanii* (ATCC 30010) was cultured in peptone yeast glucose (PYG) medium [2 % bacto proteose peptone 2, 0.1 % yeast extract, 0.1 % sodium citrate dihydrate, 0.4 mM CaCl_2_, 4 mM MgSO_4_.7H_2_O, 2.5 mM Na_2_HPO_4_.7H_2_O, 2.5 mM KH_2_PO_4_, 0.05 mM Fe(NH_4_)_2_(SO_4_)_2_.6H_2_O; 100 mM glucose, pH 6.5] in flasks at 30 °C. For infections, the amoeba culture was seeded in 24-well plates with 5×10^5^ *A. castellanii* cells with 1 ml of PYG culture. The plates were left to incubate for 1 h at 30 °C, after which the PYG was removed, and 1 ml of PYG special (PYG without peptone, yeast extract and glucose) was transferred to each well of 24-well plates. The plates were left to incubate for another hour, after which the medium was removed and replaced with 1 ml of fresh PYG special medium, to ensure proper washing of the host cell.

Human monocyte-like U937 cells (ATCC-CRL-3253) were maintained in RPMI 1640 + GlutaMAX (Gibco) supplemented with 10 % heat inactivated FBS (Gibco) and 1 % penicillin-streptomycin (PS) (Gibco), in a 37°C incubator with 5 % CO_2_. Before infection, cells were harvested, and the viability and density were assessed using 0.4 % Trypan blue solution (Gibco) and an automated cell counter (Countess FL, ThermoFisher). The cells were then centrifuged at 200 *g* for 5 min, and resuspended in growth medium with 50 ng ml^−1^ of phorbol 12-myristate 13-acetate (PMA) for 48 h to induce differentiation into macrophage-like cells. The medium was then replaced with fresh medium and cells incubated for a further 48 h. On the day of infection, the medium was changed to infection medium: RPMI 1640 without phenol red (Gibco) supplemented with 10 % heat inactivated FBS and 1 % GlutaMAX (Gibco); this medium also does not support *Legionella* growth.

### Extracellular growth assays

*L. pneumophila* strains were grown on CYE plates at 37°C for 48 h, resuspended in AYE, and diluted to an absorbance at OD_600_ of 0.1 (ca. 2×10^8^ c.f.u.). In a 96-well plate, 200 µl (ca. 4×10^7^ c.f.u.) of each cell culture was aliquoted per well (five replicates each). Growth rate was tracked by measuring OD_600_ every 30 min for 72 h at 37°C, and 180 rpm shaking, using a Tecan Spark.

### Infection assays

*L. pneumophila* infection assays were conducted in the amoeba species *A. castellanii*, and in U937 differentiated macrophages, at an m.o.i. of 0.1 and 10. Both infections were done in triplicate.

On the day of infection, the plates containing *A. castellanii* and U937 cells were inoculated with the different *L. pneumophila* strains at the corresponding m.o.i. (5×10^4^ c.f.u. for m.o.i. 0.1; 5×10^6^ c.f.u. for m.o.i. 10). Infection plates were incubated at 30 and 37 °C, respectively, with 5 % CO_2_, for 4 days. The c.f.u. counts were taken every day by plating on CYE agar plates.

*L. pneumophila* Paris strain and an *L. pneumophila* Paris ∆DotA mutant (kindly provided by Dr Elisabeth Kay, University of Lyon) were used as a positive and negative control, respectively, for the infection assays.

### Growth rate analysis

Growth rates were calculated by importing the OD data into R [45] and analysed with Growthcurver v0.3.1 with default settings [56]. Growthcurver estimates growth rates by fitting the data into a simple logistic equation.

## Results

### Isolate pairing

We sequenced the genomes of 156 clinical and environmental samples belonging to *L. pneumophila* using the Illumina MiSeq platform. These strains were isolated from clinical and environmental sources sampled during the investigation of sporadic cases in Sweden and France, as well as from outbreaks in Madrid (1996) [57] and Murcia (2001) [58], Spain. Most strains corresponded to serogroup 1, but sequence types varied (Table S1). Additional samples, including genomic data, were retrieved from published outbreak investigations (Table S2) [20, 24, 27–31, 59]. Each clinical sample was paired to its environmental relative, to form a comparison. In the few cases where several environmental samples were available, the one with the fewest SNPs as compared to the clinical strain was selected as the reference. Comparisons potentially caused by co-infections or independent infections within a short time period were removed from our final analysis [27, 60, 61]. After filtering, we obtained 171 comparisons (Tables 1 and S3), of which 100 come from isolates sequenced in this study, and 71 from publicly available data. In total, 24 comparisons come from two separate outbreaks and 147 from isolated Legionnaires’ disease cases.

**Table 1.** Summary of the comparisons between environmental and clinical strains analysed in this study

Sample origin	Comparisons
Madrid 1996 outbreak	7
Murcia 2001 outbreak	17
Folkhälsomyndigheten	18
National Legionella Reference Centre, Lyon	58
Published	71
**Total**	**171**

**Fig. 1.**
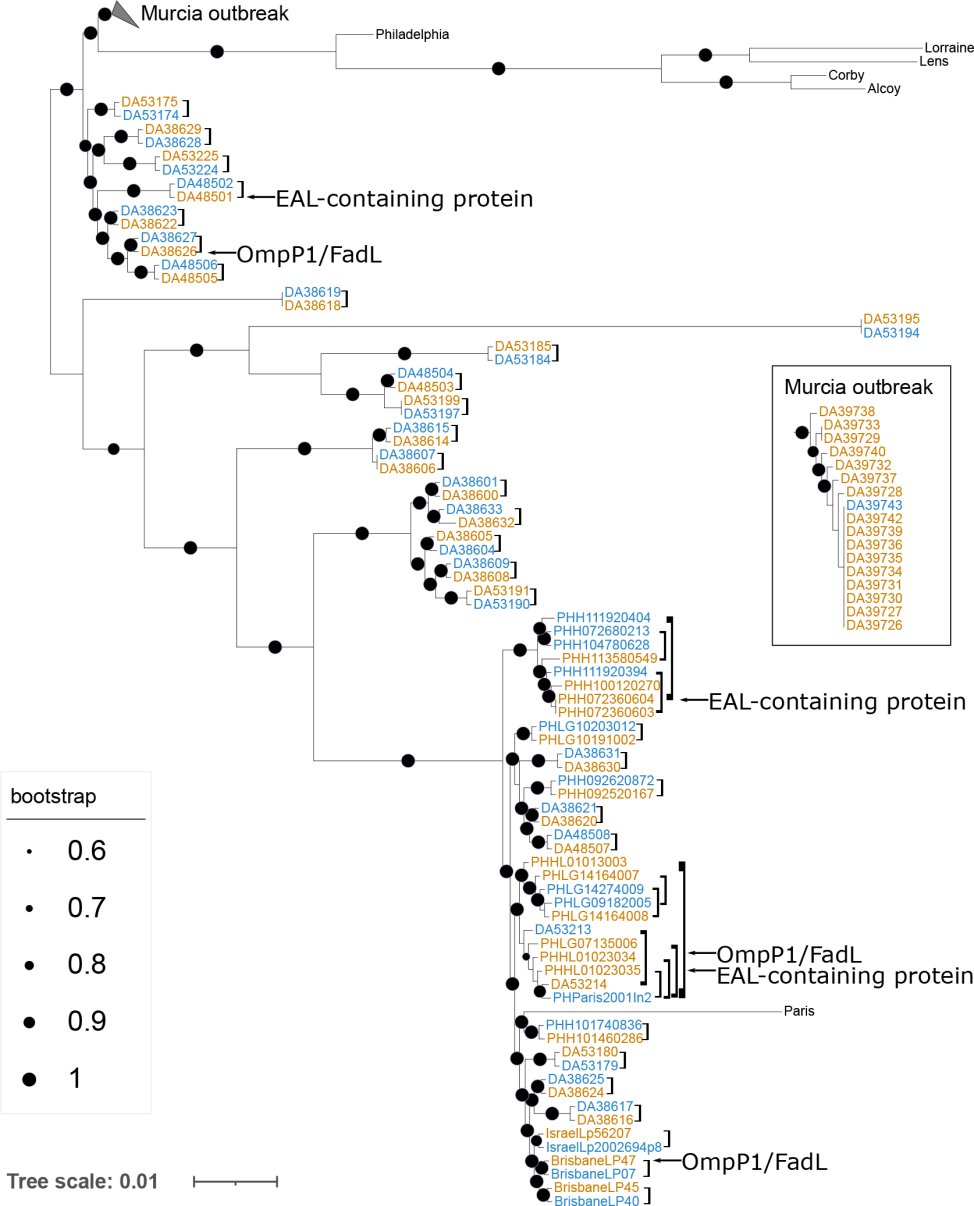
Unrooted maximum-likelihood phylogenetic tree of *L. pneumophila *. All samples in this study, as well as a few reference ones (strains Paris, Philadelphia, Lorraine, Lens, Corby and Alcoy) are included. Strains from the Murcia outbreak are collapsed for readability and shown in an inset. Environmental isolates are shown in blue, and clinical isolates in orange. Comparisons from single cases are shown with brackets. Samples containing mutations in the EAL-containing protein and samples containing mutations in the OmpP1/FadL protein are indicated by arrows. The tree is based on a *Legionella *-specific cgMLST scheme, resulting in an alignment of 2 253 410 nt positions. Circles on branches represent the percentage of bootstrap trees supporting the node. Bootstrap support values <60 are not shown. Bar, average number of substitutions per site.

A phylogeny based on the core genome shared by 95 % of all samples confirmed the similarity between the pairs of samples and among the isolates from outbreaks (Fig. 1). Supplementation of the phylogeny with strains Alcoy, Corby, Lens, Lorraine, Paris and Philadelphia revealed that many strains are closely related to *L. pneumophila* Paris.

### Genomic variation between pairs of isolates

To investigate what genetic variants might have occurred or been selected in-patient, reads from environmental isolates were assembled *de novo* and reads from the corresponding clinical strain were mapped to the assembled environmental isolate. Comparisons considered in this study had at most 20 substitutions. In 16 comparisons, no differences could be found. In total, 206 substitutions (173 SNPs and 33 short indels) were identified in the 171 comparisons. Among indels, two (6 %) were in-frame. Among the 173 SNPs, 123 (71 %) were non-synonymous and the remaining 50 (29 %) were synonymous; in five (3 %) of the cases, the substitution resulted in a premature stop codon. Ninety (52 %) were transitions and 83 (48 %) were transversions.

### Simulations

**Fig. 2.**
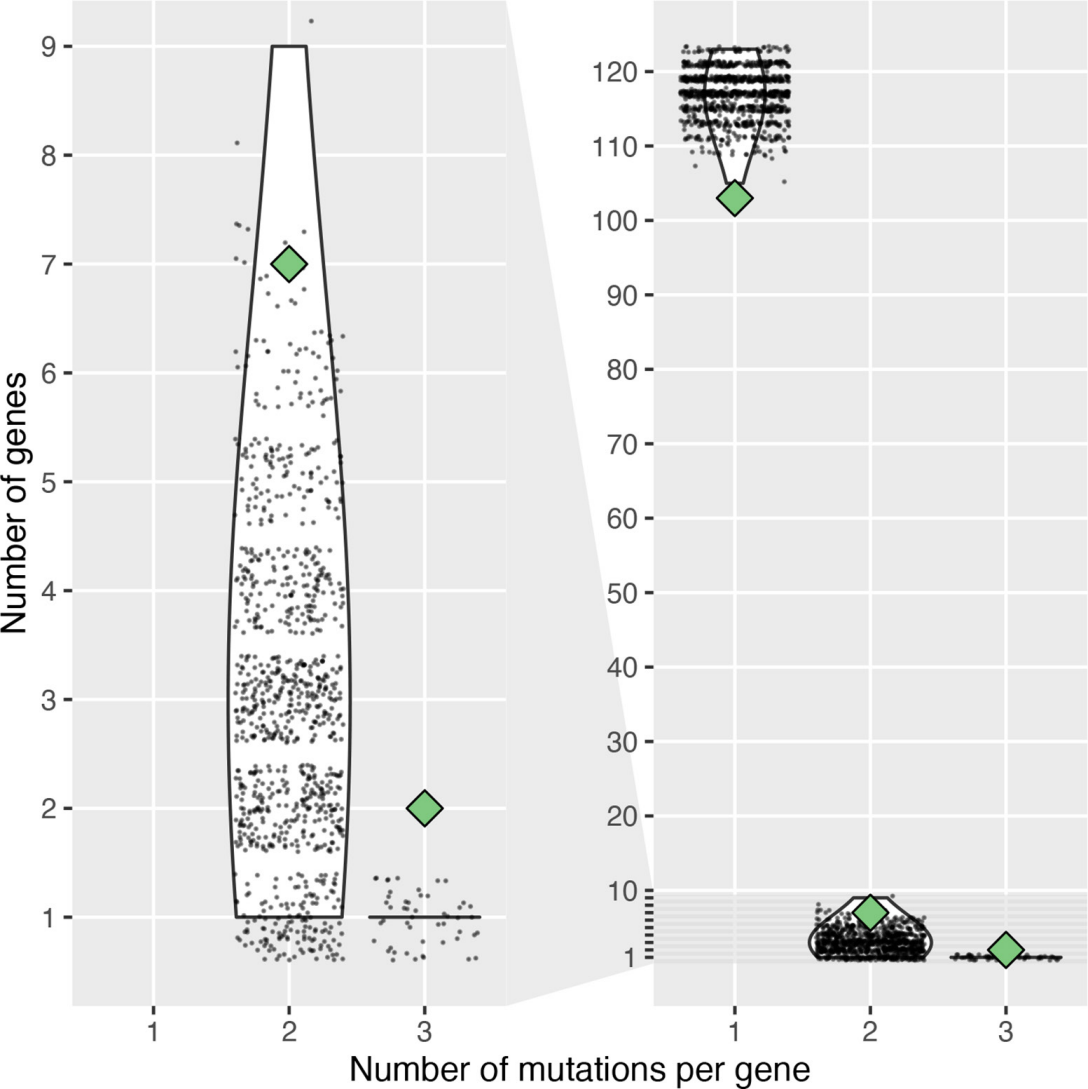
Number of mutations per gene in this study compared to 1000 simulations based on random sampling. The left panel is a subset of the right panel. The numbers of genes mutated once or more in comparisons between an environmental and clinical isolate observed in this study are displayed with green diamonds (103 genes mutated once, seven mutated twice, two mutated three times). The distribution of the number of genes mutated the same number of times in 1000 random samplings of 123 SNPs among an estimated 2445 non-essential genes of *L. pneumophila* strain Paris is displayed with violin plots. Individual data points are overlaid as a cloud of points.

To simulate the number of SNPs per gene that could be expected in an environment without selective pressure, 1000 studies were simulated, where 123 SNPs (total number of intragenic non-synonymous SNPs found in this study) were randomly distributed to the genes of *L. pneumophila* strain Paris. We then compared the simulated distributions to the observed results (two genes with three mutations, seven with two mutations, 103 with one mutation; see below). To account for genes that are under very strong negative selection and cannot accept mutations, we excluded essential genes, using a conservative estimate of 588 essential genes [42]. We observed the random distribution of 123 SNPs among the remaining 2445 non-essential genes (Fig. 2). Among the 1000 simulations, only 20 showed seven or more genes mutated twice, while none had two genes mutated three times. The probability of obtaining seven or more genes mutated twice, and of two genes mutated three times, from the simulated random distribution was estimated by one-sided Mann–Whitney *U* tests, using a single value as one of the samples. Seven genes or more mutated twice was significantly more than the distribution obtained by random sampling (*P*=0.042), although only marginally. On the other hand, two genes mutated three times was significantly more than expected from the random distribution (*P*<10^−6^).

We repeated the simulation using a very conservative estimate of only 1000 genes able to accept mutations. The probability of having two genes mutated three times was still significantly higher than by chance (*P*=0.00020). These results indicate that substitutions are not distributed randomly, but that there is an excess of genes mutated multiple times, suggesting that convergent and/or parallel evolution is at play.

### Parallelly mutated genes

To investigate which genes were mutated in multiple independent infections or outbreaks we linked our variants to orthologous gene families obtained with OrthoMCL. Variants occurring in identical families in two or more comparisons, and therefore likely to represent instances of parallel evolution, can be seen in Table 2. Although the individual substitutions are, formally at the molecular level, parallel (e.g. in lpg2009), convergent (e.g. in lpg0707) or independent (e.g. in lpg1096), we refer to the process at gene level as parallel evolution, since the mutated genes are homologous.

**Table 2.** Genes mutated more than once in clinical strains of *L. pneumophila
*

Gene	Locus tag/accession	*N*†	Environmental isolate	Clinical isolate	Substitution‡
EAL domain-containing protein	lpg0891	3	PHH111920404	PHH072360604	G662R
			PHParis2001In2	PHHL01023035	G157D
			DA48502	DA48501	I62T
OmpP1/FadL protein	lpg0707	3	BrisbaneLP07	BrisbaneLP47	G26*
			PHParis2001In2	PHHL01023034	W245*
			DA38627	DA38626	G311D
Efflux RNS transporter permease subunit	lpg1096	2	IsraelLp2002694p8	IsraelLp56207	G774D
			DA38605	DA38604	F157F
KaiB-like protein 2	WP_027224181.1	2	DA38609	DA38608	N359I
			DA53224	DA53225	338_339del
DUF1566 domain-containing protein	WP_014844754.1	2	DA38601	DA38600	W423*
			DA38633	DA38632	372_373insGTG
GTP diphosphokinase	lpg2009	2	DA53179	DA53180	G113V
			DA53213	DA53214	G113V
Glycosyltransferase family 2 protein	lpg2478	2	DA38605	DA38604	R139S
			DA38601	DA38600	42del
ATP-dependent helicase/deoxyribonuclease subunit B	lpg2269	2	PHLG09182005	PHLG14164007	S413N
			PHLG14274009	PHLG14164008	S413N
CAAX amino terminal protease	lpg1525	2	DA38605	DA38604	C153CA
			DA38609	DA38608	AGG1AG

†Number of substitutions in this gene.

‡Amino acid substitutions are represented by the original amino acid, the position of the substitution, and the new amino acid. Asterisks represent stop codons. Indels are shown with the position of the insertion/deletion, ins for insertions and del for deletions, and the inserted sequence for insertions.

Of particular interest are genes mutated independently in three separate comparisons: one encodes an (outer) membrane protein (lpg0707/lpp0762/lpl0744), and the other one an EAL domain-containing protein (lpg0891/lpp0952/lpl0922). The outer membrane protein belongs to the OmpP1/FadL family. Two of the mutations led to proteins being respectively 97 and 60% shorter than the wild-type protein by introducing a stop codon, while the third mutation did not lead to a change in secondary structure. The EAL-containing domain protein was also mutated three times, but none of the three mutations resulted in a shortened protein. These two genes are referred to as potentially adaptive in the human host (PAHH). The sequence of these variants is available in Supplementary Data 1.

We searched all published *L. pneumophila* proteomes for the specific mutations in the EAL-containing protein and in OmpP1/FadL. The mutations in the EAL-domain-containing protein were specific to our strains and could not be found in any other published *L. pneumophila* strain. On the other hand, we identified four different non-sense mutations that yielded shorter OmpP1/FadL proteins. These mutations were present in 33 proteins out of the 2737 close OmpP1/FadL homologues (1.2 %). The metadata associated with these genomes did not allow us to reliably establish whether the isolates they come from are clinical or environmental.

#### Growth assays

To determine the *in vitro* effect of the PAHH variants, two clinical strains (DA38626 and DA48501), their respective environmental strains (DA38627 and DA48502), together with *L. pneumophila* Paris (DA57510) as a reference strain and an intracellular growth-deficient mutant (lacking DotA) were cultivated in growth medium and in the presence of two hosts, the amoeba *A. castellanii* (Acas) and the human-macrophage derived U937 cells. DA38626 carries a mutation in the OmpP1/FadL homologue, while DA48501 carries one in the EAL-containing protein. The corresponding environmental strains are otherwise isogenic to the clinical strains, although we cannot exclude the possibility that some structural variations were missed, due to the sequencing method used (Illumina).

To assess the effect of the PAHH mutations, the two pairs of isolates were first grown extracellularly, in liquid medium (Fig. S1). The mutation in the OmpP1/FadL homologue in DA38626 resulted in a very small difference in growth rate (2.1 % lower for DA38626, resulting in a 3.3 min difference in doubling time). The mutation in the EAL-containing protein resulted in a more important reduction of growth rate (16.9 % lower growth rate, resulting in a 21.4 min increase in doubling time). The reference Paris strain (DA57510) displayed an intermediate growth rate (0.41, doubling time of 100 min).

**Fig. 3.**
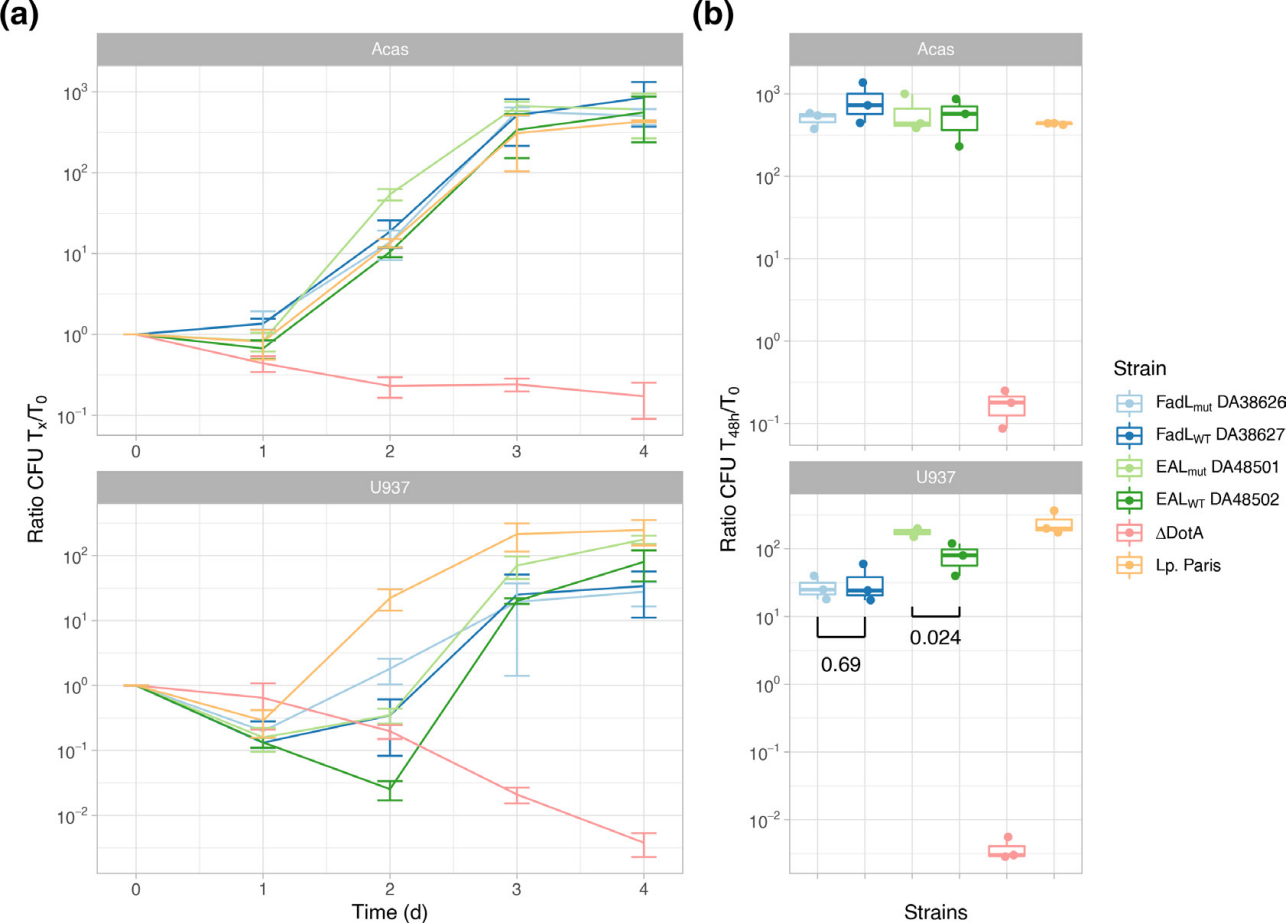
Intracellular replication of *L. pneumophila* in *A. castellanii* (Acas) and human macrophage-derived U937 cells at an m.o.i. of 0.1. DA38626 (clinical isolate, pale blue) carries the mutated OmpP1/FadL homologe (lpg0707), while DA38627 (environmental, dark blue) has the wild-type gene. DA48501 (clinical isolate, pale green) carries a mutated EAL-containing protein (lpg0891), while DA48502 (environmental, dark green) has the wild-type gene. Two controls are shown: *L. pneumophila* strain Paris wild-type (Lp. Paris, orange) and a DotA mutant of the same strain, deficient for intracellular growth (ΔDotA, red). (**a**) Growth, as measured by the c.f.u. count ratio relative to T_0_, over time (*x*-axis, in days). Each curve shows the average of three replicates. Error bars show standard deviation. (**b**) Ratios of c.f.u. counts after 2 days (T_48h_) compared to c.f.u. counts at T_0_. Each dot represents a replicate, and box-and-whiskers plots summarize the data. *P*-values of two-sample *t*-tests are shown for the comparison between the mutant and wild-type alleles, for both the OmpP1/FadL gene and the EAL-containing protein. Top panels show growth in *A. castellanii* (Acas), while bottom panels show growth in human macrophage-like U937 cells.

Intracellular replication growth was then estimated by infecting *A. castellanii* and U937 cells with the selected strains, at two different multiplicity of infection (0.1 and 10) and following intracellular growth over 4 days. Growth curves (Figs 3 and S2) indicate no significant growth difference of the OmpP1/FadL-variant bearing clinical isolate DA38626 compared to the environmental isolate DA38627, either in *A. castellanii* or in macrophages (*P*=0.69 for the latter comparison).

The EAL variant-bearing clinical isolate DA48501 compared to the environmental isolate DA48502 also showed similar growth in *A. castellanii* cells but, contrastingly, a significantly increased growth after 4 days in macrophages (*P*=0.024), despite its lower growth rate in liquid medium. Interestingly, compared to all these isolates, the control *L. pneumophila* Paris grows slightly slower in *A. castellanii* but faster in U397 cells. At a m.o.i. of 10, strain DA38626 encoding the mutated OmpP1/FadL protein displayed similar growth in *A. castellanii*, but, surprisingly, a reduced growth in macrophages (Fig**.** S2). On the other hand, the clinical strain DA48501 carrying the EAL-containing protein variant also displayed a higher growth at a higher m.o.i., although the difference was not significant.

## Discussion

By comparing a large number of *L. pneumophila* clinical isolates from outbreaks and sporadic cases to their environmental source we were able to identify nine genes, which acquired a mutation in more than one independent case. Of these, two genes were mutated three times. Mutations in these genes were probably selected in-patient, even though the mutation itself might have occurred prior to the infection. These genes showing signs of parallel evolution potentially represent adaptations of *L. pneumophila* to the human host.

These mutations presumably come mostly from point mutations, but another driver of genomic diversity is horizontal gene transfer (HGT). In *L. pneumophila *, both conjugation and transformation have been described [21, 22, 62]. Recombination events are frequent and the exchange of large fragments (>200 kb) has been described [23]. In addition, within certain lineages 95 % of SNPs arose due to recombination events [20, 25]. While stretches of mutations originating from recombination were found in our own samples, they did not account for any of the possible candidates of host adaptation. Although some of the isolated mutations included in our analysis might still come from recombinations, these would be from recombinations between very closely related strains, and do not affect the results of our analysis. Indeed, mutations resulting from recombinations may still represent adaptations.

Another complicating factor may have been the time between infection and environmental sampling [63], which could have led to the rise of variants in the environmental samples. By obtaining enough samples, and by removing comparisons from outbreaks in which the identical mutation occurred in all comparisons, this effect is limited. Additionally, for both genes mutated three times, we always observed the same wild-type allele in the environmental strain and a mutated one in the clinical strain.

The probability of obtaining as many as seven genes independently mutated without selection is low, but genes mutated twice appeared in all simulations. Since it is not possible to discriminate with high confidence, among the seven candidates, those that arose by chance alone from occurrences of parallel evolution, genes mutated twice are not discussed further. We focus on the two genes mutated three times, which occur only very rarely in simulations. These two candidates, encoding an outer membrane protein (OmpP1/FadL homologue; lpg0707) in which two out of three mutations encode an early stop codon and an EAL-containing protein (lpg0891), are discussed below. All mutations occurring in these two genes have presumably occurred either in-patient or slightly before in the same strain. Even mutations that occurred before infection in the direct ancestor of the strain that eventually infected the patient are relevant, since those mutations have a selective value and favour the infection.

**Fig. 4.**
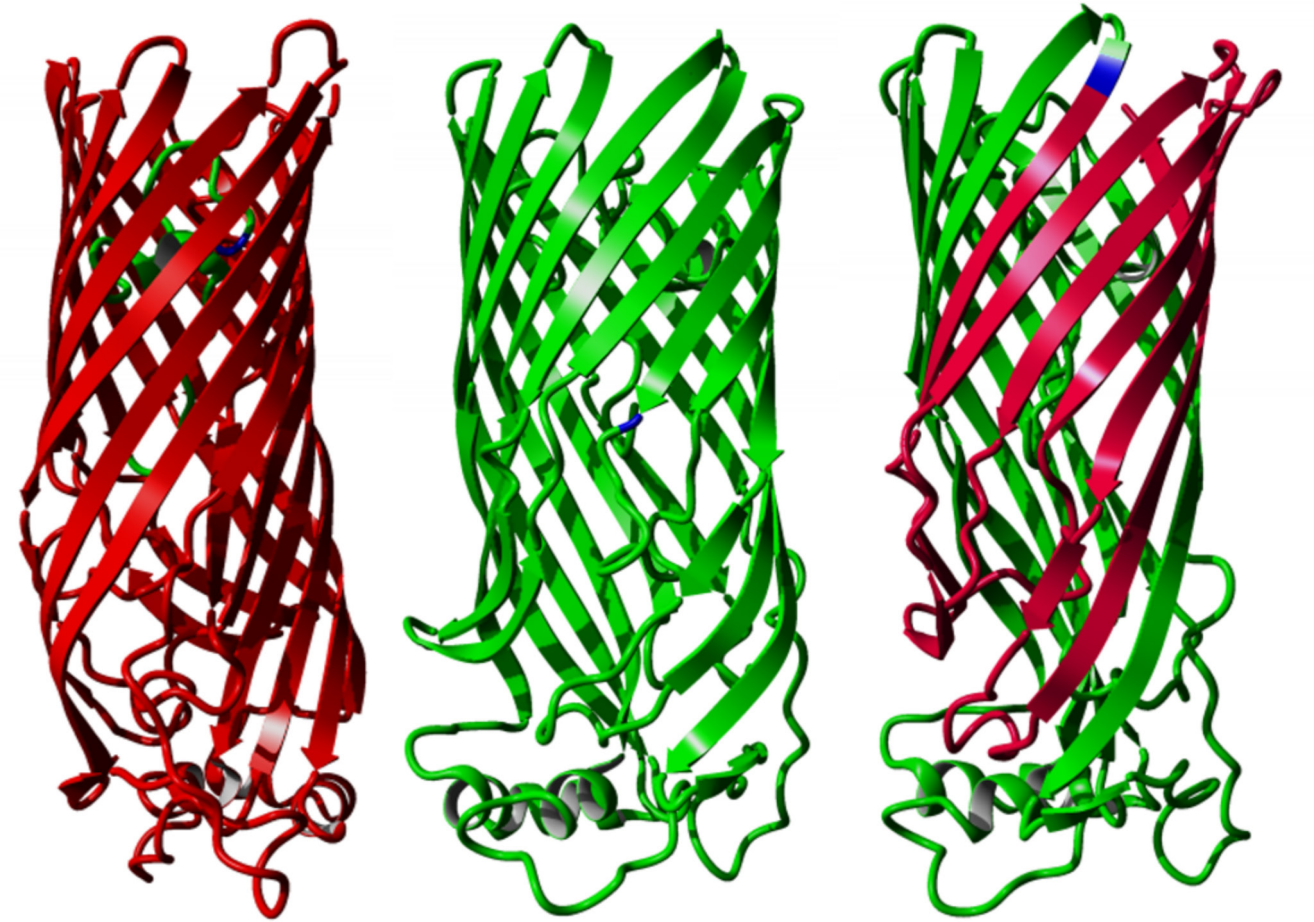
Predicted structure of *Pseudomonas aeruginosa* FadL homologue (PDB: 3DWO), with the corresponding location of the variants highlighted in blue (left: strain BrisbaneLP47, G26*, aligned to S28 in 3DWO; middle: strain PHHL01023034, W245*, aligned to L300 in 3DWO; right: DA38626, G311D, aligned to T362 in 3DWO). Red indicates the part of the protein that would not be translated after the introduced stop codons.

In addition to LPS and phospholipids, around 250 proteins populate the *L. pneumophila* outer membrane [64]. Most of their functions remain unclear, and this includes the outer membrane protein encoded by the gene lpg0707 identified in this study. This protein belongs to the OmpP1/FadL family, which is conserved throughout bacteria and is required for the uptake of hydrophobic long-chain fatty acids via lateral diffusion through the outer membrane [65, 66]. In this study, two of the mutations cause a premature stop codon at amino acid positions 26 and 245. Based on a protein sequence alignment and the structure of a *Pseudomonas aeruginosa* FadL homologue [66], these stop codons would disrupt the β-barrel that functions as a hydrophobic tunnel through which substrates pass (Fig. 4), which is likely to lead to a non-functional protein. The third mutation replaces a glycine (small, non-polar) with a negatively charged aspartate at position 311, in the part located in the middle of the hydrophobic membrane. Although the exact effect of the latter mutation cannot be determined without further experimental evidence, it is not unreasonable to believe that it affects the function of the OmpP1/FadL protein in a significant way, possibly also resulting in a loss-of-function mutation.

Our growth assays show that the mutation in OmpP1/FadL does not confer significantly higher growth in the human macrophage cell line U937. While this result is surprising, it might be explained by the fact that the selective pressures that favoured the mutant allele in the three independent infections happened outside of the cell. One hypothesis is that OmpP1/FadL, located on the outside of the *Legionella* cell, might be recognized by the host immune system, which is not included in our experiment. In a screening of *Salmonella paratyphi A* CMCC 50973 outer membrane protein, FadL was found to be strongly immunogenic and thus proposed as a candidate for a vaccine [67]. Therefore, modifications or disruptions to proteins located in the outer membrane could allow *L. pneumophila* to avoid early detection by the human host’s immune system, and allow it to establish itself inside the alveolar macrophages.

EAL domain-containing proteins regulate cyclic diguanylate (c-di-GMP), which modulates pathways involved in life cycle transitions, e.g from pathogenic to environmental lifestyles, or from planktonic infections to biofilm infections [68–72]. Cyclic-di-GMP is produced from GTP by diguanylate cyclases (DGCs), and is degraded via phosphodiesterases (PDEs). The catalytic site of DGCs is identified as a GGDEF domain, while PDE activity involves either an EAL or the HD-GYP domain [73–75]. *L. pneumophila* encodes 22–24 GGDEF/EAL proteins, and most are highly conserved in *L. pneumophila* strains, suggesting an important role of GGDEF/EAL domain proteins for regulation of the *L. pneumophila* life cycle [72, 76]. Interestingly, one of the other EAL domain-containing proteins (lpp0942/lpg0879) displayed parallel mutations in four of the five branches leading to disease-associated sequence types (STs), also qualifying as a potential human-specific adaptation [25]

**Fig. 5.**
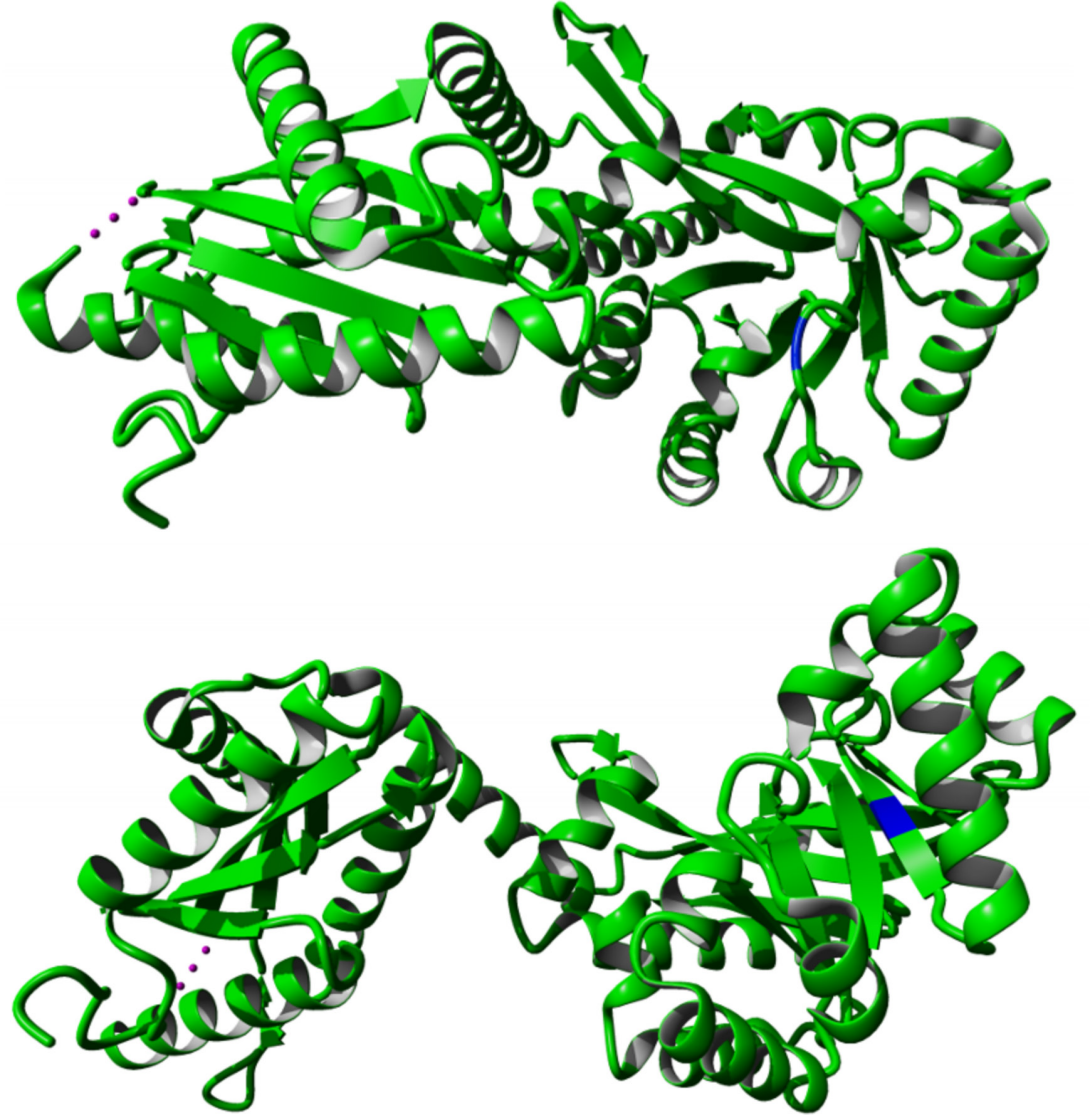
Predicted structure of *Pseudomonas aeruginosa* MorA homologue (PDB: 4RNH), with variants highlighted in blue. Top: G662D mutation in strain PHHL01023035, aligned to G1315. Bottom: I711T in strain DA48501, aligned to I1365. The G157R mutation in strain PHH072360604 does not align to the crystallized part of MorA and is thus not shown here.

Upon infection, *Legionella* uses its Dot/Icm T4SS to secrete over 300 effector proteins to establish a vacuole called the *Legionella *-containing vacuole (LCV), in which it may replicate, until nutrients become depleted. Nutrient depletion triggers the expression of virulence factor-encoding genes, and the upregulation of genes containing GGDEF/EAL domains [77–79]. The specific protein encoded by lpg0891, mutated independently three times in this study, harbours a DGC domain and PDE domain, both functional [80]. Two out of three mutations occurred within an EAL domain (Fig. 5), but none apparently resulted in a loss-of-function mutation. All three mutations replace a non-polar amino acid by a polar one. In strain Philadelphia, the protein was shown to be expressed only in the post-exponential phase, i.e. at the end of the intracellular replication, when *Legionella* is virulent and motile [81]. In the same strain, knockout mutants for this protein are still able – albeit to a slightly lesser extent – to grow in BYE broth, and to infect both amoebae and macrophages, to translocate and to evade lysosomes [80]. This observation is consistent with our EAL mutant – albeit probably still functional – growing slower in liquid culture. Furthermore, experiments in strain Lens have demonstrated that the homologue protein encoded by lpl0922 is crucial for setting up efficient intracellular replication, both in amoebae and in macrophages [82]. It has also been shown that the homologue protein in strain Paris (encoded by gene lpp0952) plays an important role in the regulation of flagellar activity and of motility, and is regulated by the flagellar regulator FleQ, presumably via FliA [83].

The EAL-domain-containing protein mutant studied here displays a similar growth rate as the wild-type in the amoebal host, but a significantly higher growth when infecting human macrophage cell lines, at least at low m.o.i. This suggests that the mutant is better adapted to human macrophages, without a clear cost to its ability to infect and replicate in its natural amoebal host. Further investigations are required to determine the exact mechanism by which mutations in the EAL-containing protein provide an advantage while infecting human macrophages. However, the various infection-related processes in which it is involved (see above) provide interesting starting points to explore its function in human cells.

A recent extensive study by Park *et al.* [26] systematically explored the importance of *L. pneumophila* genes for growth in amoebal hosts – including *A. castellanii* – and the human host (using the same U937 cell line as in this contribution), in a transposon sequencing approach. Their study highlighted that different hosts impose different evolutionary constraints on *Legionella *, with genes or gene variants being beneficial in some hosts and detrimental in others. Of particular interest, the OmpP1/FadL (lpg0707) mutants analysed by Park *et al.* displayed a similar fitness to the wild-type strain in *A. castellanii*, but a slightly decreased one in U937 hosts, in line with our results where the knockout mutant grew slightly worse in U937 cells at high m.o.i. (Fig. S2). Regarding the EAL-containing protein mutant (lpg0891), it also showed a similar fitness to the wild-type in *A. castellanii* but an increased fitness in U937 in the Park *et al.* study. This result is also consistent with our results, where we show that our EAL-containing protein mutant displays a higher growth in U937 cells.

The human host is generally considered as an evolutionary dead-end for *L. pneumophila *, since human-to-human transmission is very rare and human-to-environment transmission routes are unknown. However, the independent emergence in the past century of five widespread, disease-associated STs has prompted authors to suggest that these STs have adapted to human-made water systems [25]. Furthermore, the same authors hypothesize that those STs, which are apparently not overrepresented in human-made water systems, might be adapted to the human host (and not only to its water systems) and spread via human-to-human and/or human-to-environment transmission [25]. In our study, for each of the two genes of interest (EAL-containing domain protein and OmpP1/FadL) two of the three mutations occurred in ST1, one of the five STs identified as potentially adapted to the built environment. Thus, even if these lineages are indeed adapted to the built environment, they still harbour genes that, once mutated, become selected in-patient, indicating that they are not very well adapted to the human host.

In summary, we have identified two potential candidate genes for human-specific adaptation in *L. pneumophila *. This included a regulatory protein able to both synthesize and degrade cyclic-di-GMP, which is involved in the virulence and motility of *Legionella *, and provided *L. pneumophila* with an increased growth in human macrophages. We also identified signs of parallel evolution in an OmpP1/FadL outer membrane protein, in which mutations led to several premature stop codons. The repeated detection of mutation in the same genes, occurring during the short time (2–14 days) of the onset of the disease in patients or prior to the infection and then selected in-patient, is consistent with these mutations being beneficial to *L. pneumophila* when infecting human hosts. This suggests that *L. pneumophila* is not as well adapted to the human host than to amoebal hosts.

## Supplementary Data

Supplementary material 1

Supplementary material 2
