# Supplementary material for: Rapid adaptations of Legionella pneumophila to the human host
Source: Microb Genom. 2023 Mar 22;9(3):mgen000958. doi: 10.1099/mgen.0.000958 (PMC10132064; doi:10.1099/mgen.0.000958)
Supplement: Supplementary material 1 [file mgen-9-958-s001.pdf]

Rapid adaptations of *Legionella pneumophila* to the human host

Daniël Leenheer, Anaísa B. Moreno, Kiran Paranjape, Susan Murray, Sophie Jarraud, Christophe Ginevra, Lionel Guy

Supplementary Figures

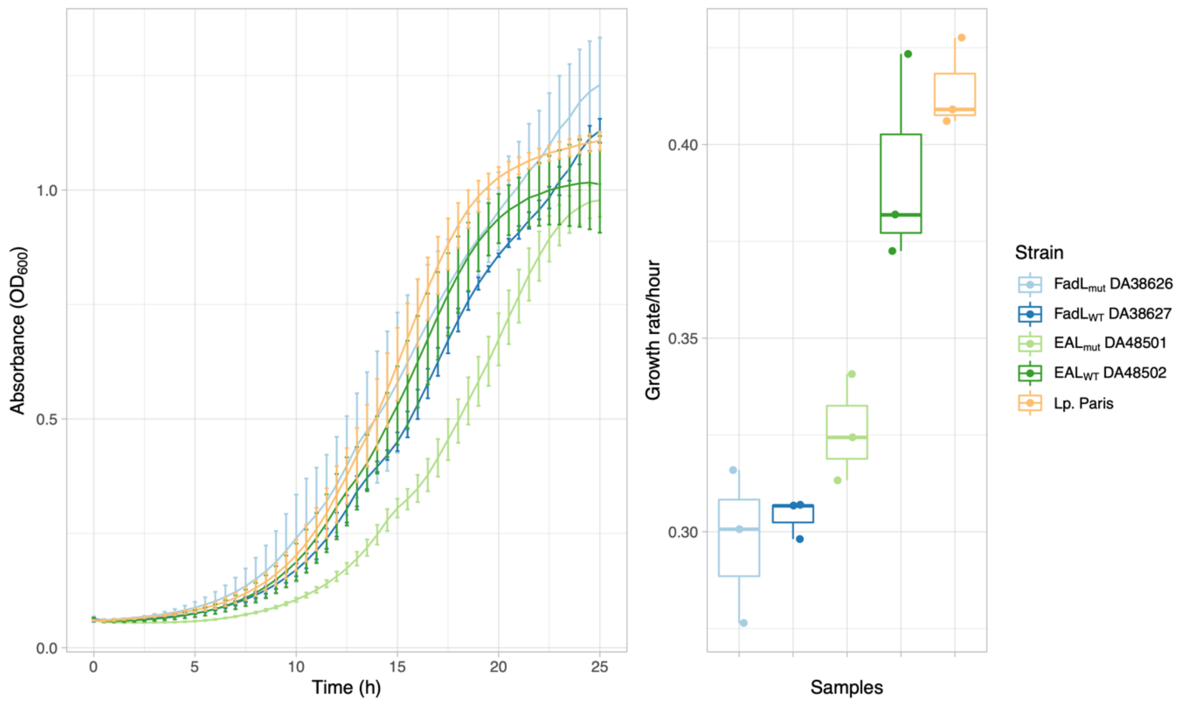

Supplementary Figure 1. Growth in liquid medium of the two strains carrying a potential adaptation to the human host, compared to their wild types, measured by absorbance at 600 nm. On the left panel, each curve is the average of three technical replicates. Standard deviation is shown at each time point. The right panel, the growth rate estimated by fitting a logistic equation to the growth curvres. Each replicate is shown by a dot, and the data is summarized by a box-and-whiskers plot. DA38626 (clinical isolate, pale blue) carries the mutated OmpP1/FadL homolog (lpg0707), while DA38627 (environmental, dark blue) has the wild type gene. DA48501 (clinical isolate, pale green) carries a mutated EAL-containing protein (lpg0891), while DA48502 (environmental, dark green) has the wild type gene. *L. pneumophila* str. Paris (orange) serves as control.

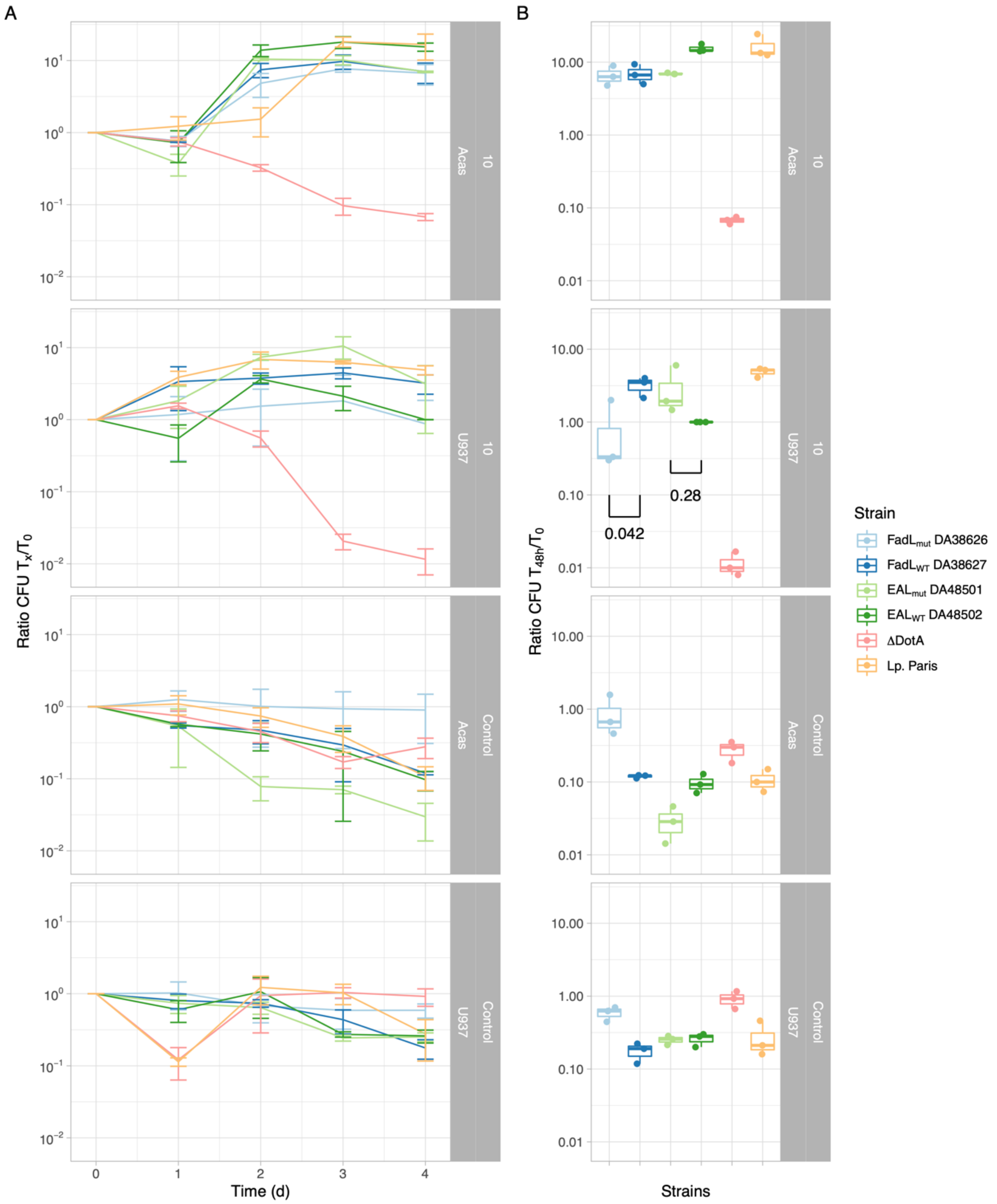

Supplementary Figure 2: Intracellular replication of *L. pneumophila* in *A. castellanii* (Acas) and U937 cells at an MOI of 10 (top two rows) and controls of growth in infection medium (bottom two rows). DA38626 (clinical isolate, pale blue) carries the mutated OmpP1/FadL homolog (lpg0707), while DA38627 (environmental, dark blue) has the wild type gene. DA48501 (clinical isolate, pale green) carries a mutated EAL-containing protein (lpg0891), while DA48502 (environmental, dark green) has the wild type gene. Two controls are shown: Lp. Paris (orange) and ΔDotA (red) are *L. pneumophila* str. Paris wild-type (Lp. Paris,

orange) and a DotA mutant, deficient for intracellular growth (ΔDotA, red). The top two rows show growth in *A. castellanii* (Acas) and growth in human macrophage-like U937 cells, respectively, at MOI 10. The bottom two rows show growth of the strains in the medium used for infection in Acas and U937, respectively. **A:** Growth, as measured by the CFU count ratio relative to T_0, over time (x-axis, in days). Each curve shows the average of three replicates. Error bars show standard deviation. **B:** Ratios of CFU counts after two days (T_{48h}) compared to CFU counts at T_0. Each dot represents a replicate, and box-and-whiskers plots summarize the data. P-values of two-sample t-tests are shown for the comparison between the mutant and wild-type alleles, for both the OmpP1/FadL gene and the EAL-containing protein.

Supplementary Tables

Supplementary Table 1

Isolates sequenced in this study. Separate Excel file.

Supplementary Table 2

Strains obtained from published data. Separate Excel file.

Supplementary Table 3

Comparison clinical-environmental isolates analyzed in this study. Separate Excel file.

Supplementary Data

Supplementary Data 1

Protein sequences of the genes mutated three times

```
>DA48502 EAL-containing protein, environmental isolate
MRRQEINYEKVAKAAEKIKKRAIEPSVNEIRDELGLVGNHPQLSILLEEWYHNQPEFKRK
SITPLTENINLNTDEIREKNVELEKSISLLRATLESTADGIMMVNGHGAVVDWNQKFVEM
WRIPSYMMESGKESISFEYILEQLIDPQSLIADVQCLYQNPEWQGELPELHFKDGRIYER
FTQPQRVGSQIVGRVYSFRDVTQKRMALDELRIRERAIEASTHGVVIIDVTKNENKVIYV
NRAFERITGYGEQHALGKGLLTLLGSNLEEVNHKRIELAIRESKEETIEMESIKRNGEFY
WCEISVAPVKDSFGYVKHYICILNDVTQRRDMEDQLLLQATYDSLTNLPNRVLLMDRVEQ
AILQARKNKAILAFLFLDLDRFKLTNDTLGHSMGDKLLQAIANRLLIVTEDFDTVARLGG
DEFVILLTDIDNMLEAETIAQNILKIIEKPIQIDQHSLKITGSLGISFYPRDGDDYESLM
KSADLSMYHAKDTGRNNYRVYEPEMNRRVINHMQLDNALRDALKNDELFLVYQPLIDLKQ
SRVVGFEALMRWHSKILGLVSPADFIPMAEENGMILEMGEWAMKQACIQVKEWHKAGFKN
LSIAVNLSGRQFRQKNLPEVVSRVLKSSGLQSRFLELELTESLLIEDIDHVVDTMYALKD
MGTKLVIDDFGTGYSSLSYLKQFPVDKLKIDRSFITEMVSNQNDAAIAKAIINLGHSLNL
```

QVLAEGVENEFQRDFITSHGCDYAQGYFFKAPDTPENILEFLKSLSESKIK

>DA48501 EAL-containing protein, clinical isolate
MRRQEINYEKVAKAAEKIKKRAIEPSVNEIRDELGLVGNHPQLSILLEEWYHNQPEFKRK
STTPLTENINLNTDEIREKNVELEKSISLLRATLESTADGIMMVNGHGAVVDWNQKFVEM
WRIPSYMMESGKESISFEYILEQLIDPQSLIADVQCLYQNPEWQGELPELHFKDGRIYER
FTQPQRVGSQIVGRVYSFRDVTQKRMALDELRIRERAIEASTHGVVIIDVTKNENKVIYV
NRAFERITGYGEQHALGKGLLTLLGSNLEEVNHKRIELAIRESKEETIEMESIKRNGEFY
WCEISVAPVKDSFGYVKHYICILNDVTQRRDMEDQLLLQATYDSLTNLPNRVLLMDRVEQ
AILQARKNKAILAFLFLDLDRFKLTNDTLGHSMGDKLLQAIANRLLIVTEDFDTVARLGG
DEFVILLTDIDNMLEAETIAQNILKIIEKPIQIDQHSLKITGSLGISFYPRDGDDYESLM
KSADLSMYHAKDTGRNNYRVYEPEMNRRVINHMQLDNALRDALKNDELFLVYQPLIDLKQ
SRVVGFEALMRWHSKILGLVSPADFIPMAEENGMILEMGEWAMKQACIQVKEWHKAGFKN
LSIAVNLSGRQFRQKNLPEVVSRVLKSSGLQSRFLELELTESLLIEDIDHVVDTMYALKD
MGTKLVIDDFGTGYSSLSYLKQFPVDKLKIDRSFITEMVSNQNDAAIAKATINLGHSLNL
QVLAEGVENEFQRDFITSHGCDYAQGYFFKAPDTPENILEFLKSLSESKIK

>PHH111920404 EAL-containing protein, environmental isolate
MRRQEINYEKVAKAAEKIKKRGIEPSVNEIRDELGLVGNHPQLSILLEEWYHNQPEFKRK
SHTPLTENINLNTDEIREKNVELEKSISLLRATLESTADGIMMVNGHGAVVDWNQKFVEM
WRIPSYMMESGKESISFEYILEQLIDPQSLIADVQYLYQNPEWQGELPELHFKDGRIYER
FTQPQRVGSQIVGRVYSFRDVTQKRMALDELRIRERAIEASTHGVVIIDVTKNENKVIYV
NRAFERITGYGEQHALGKGLLTLLGSNLEEVNHKRIELAIRESKEETIEMESIKRNGEFY
WCEISVAPVKDSFGYVKHYICILNDVTQRRDMEDQLLLQATYDSLTNLPNRVLLMDRVEQ
AILQARKNKAILAFLFLDLDRFKLTNDTLGHSMGDKLLQAIANRLLIVTEDFDTVARLGG
DEFVILLTDIDNMLEAETIAQNILKIIEKPIQIDQHSLKITGSLGISFYPRDGDDYESLM
KSADLSMYHAKDTGRNNYRVYEPEMNRRVINHMQLDNALRDALKNDELFLVYQPLIDLKQ
SRVVGFEALMRWHSKILGLVSPADFIPMAEENGMILEMGEWAMKQACIQVKEWHKAGFKN
LSIAVNLSGRQFRQKNLPEVVSRVLKSSGLQSRFLELELTESLLIEDIDHVVDTMYALKD
MGTKLVIDDFGTGYSSLSYLKQFPVDKLKIDRSFITEMVSNQNDAAIAKAIINLVHSLNL
QVLAEGVENEFQRDFITSHGCDYAQGYFFKAPDTPENILEFLKSLSESKIK

>PHH072360604 EAL-containing protein, clinical isolate
MRRQEINYEKVAKAAEKIKKRGIEPSVNEIRDELGLVGNHPQLSILLEEWYHNQPEFKRK
SHTPLTENINLNTDEIREKNVELEKSISLLRATLESTADGIMMVNGHGAVVDWNQKFVEM
WRIPSYMMESGKESISFEYILEQLIDPQSLIADVQYLYQNPEWQGELPELHFKDGRIYER
FTQPQRVGSQIVGRVYSFRDVTQKRMALDELRIRERAIEASTHGVVIIDVTKNENKVIYV
NRAFERITGYGEQHALGKGLLTLLGSNLEEVNHKRIELAIRESKEETIEMESIKRNGEFY
WCEISVAPVKDSFGYVKHYICILNDVTQRRDMEDQLLLQATYDSLTNLPNRVLLMDRVEQ
AILQARKNKAILAFLFLDLDRFKLTNDTLGHSMGDKLLQAIANRLLIVTEDFDTVARLGG
DEFVILLTDIDNMLEAETIAQNILKIIEKPIQIDQHSLKITGSLGISFYPRDGDDYESLM
KSADLSMYHAKDTGRNNYRVYEPEMNRRVINHMQLDNALRDALKNDELFLVYQPLIDLKQ
SRVVGFEALMRWHSKILGLVSPADFIPMAEENGMILEMGEWAMKQACIQVKEWHKAGFKN
LSIAVNLSGRQFRQKNLPEVVSRVLKSSGLQSRFLELELTESLLIEDIDHVVDTMYALKD
MRTKLVIDDFGTGYSSLSYLKQFPVDKLKIDRSFITEMVSNQNDAAIAKAIINLVHSLNL
QVLAEGVENEFQRDFITSHGCDYAQGYFFKAPDTPENILEFLKSLSESKIK

>PHParis2001In2 EAL-containing protein, environmental isolate
MRRQEINYEKVAKAAEKIKKRGIEPSVNEIRDELGLVGNHPQLSILLEEWYHNQPEFKRK
SHTPLTENINLNTDEIREKNVELEKSISLLRATLESTADGIMMVNGHGAVVDWNQKFVEM
WRIPSYMMESGKESISFEYILEQLIDPQSLIADVQYGYQNPEWQGELPELHFKDGRIYER
FTQPQRVGSQIVGRVYSFRDVTQKRMALDELRIRERAIEASTHGVVIIDVTKNENKVIYV
NRAFERITGYGEQHALGKGLLTLLGSNLEEVNHKRIELAIRESKEETIEMESIKRNGEFY
WCEISVAPVKDSFGYVKHYICILNDVTQRRDMEDQLLLQATYDSLTNLPNRVLLMDRVEQ
AILQARKNKAILAFLFLDLDRFKLTNDTLGHSMGDKLLQAIANRLLIVTEDFDTVARLGG
DEFVILLTDIDNMLEAETIAQNILKIIEKPIQIDQHSLKITGSLGISFYPRDGDDYESLM
KSADLSMYHAKDTGRNNYRVYEPEMNRRVINHMQLDNALRDALKNDELFLVYQPLIDLKQ
SRVVGFEALMRWHSKILGLVSPADFIPMAEENGMILEMGEWAMKQACIQVKEWHKAGFKN
LSIAVNLSGRQFRQKNLPEVVSRVLKSSGLQSRFLELELTESLLIEDIDHVVDTMYALKD
MGTKLVIDDFGTGYSSLSYLKQFPVDKLKIDRSFITEMVSNQNDAAIAKAIINLVHSLNL
QVLAEGVENEFQRDFITSHGCDYAQGYFFKAPDTPENILEFLKSLSESKIK

>PHHL01023035 EAL-containing protein, clinical isolate
MRRQEINYEKVAKAAEKIKKRGIEPSVNEIRDELGLVGNHPQLSILLEEWYHNQPEFKRK
SHTPLTENINLNTDEIREKNVELEKSISLLRATLESTADGIMMVNGHGAVVDWNQKFVEM
WRIPSYMMESGKESISFEYILEQLIDPQSLIADVQYDYQNPEWQGELPELHFKDGRIYER
FTQPQRVGSQIVGRVYSFRDVTQKRMALDELRIRERAIEASTHGVVIIDVTKNENKVIYV
NRAFERITGYGEQHALGKGLLTLLGSNLEEVNHKRIELAIRESKEETIEMESIKRNGEFY
WCEISVAPVKDSFGYVKHYICILNDVTQRRDMEDQLLLQATYDSLTNLPNRVLLMDRVEQ
AILQARKNKAILAFLFLDLDRFKLTNDTLGHSMGDKLLQAIANRLLIVTEDFDTVARLGG
DEFVILLTDIDNMLEAETIAQNILKIIEKPIQIDQHSLKITGSLGISFYPRDGDDYESLM
KSADLSMYHAKDTGRNNYRVYEPEMNRRVINHMQLDNALRDALKNDELFLVYQPLIDLKQ
SRVVGFEALMRWHSKILGLVSPADFIPMAEENGMILEMGEWAMKQACIQVKEWHKAGFKN
LSIAVNLSGRQFRQKNLPEVVSRVLKSSGLQSRFLELELTESLLIEDIDHVVDTMYALKD
MGTKLVIDDFGTGYSSLSYLKQFPVDKLKIDRSFITEMVSNQNDAAIAKAIINLVHSLNL
QVLAEGVENEFQRDFITSHGCDYAQGYFFKAPDTPENILEFLKSLSESKIK

>DA38627 OmpP1/FadL, environmental isolate
MRLIFTGVLCLLTLNAQANVIQYFAGISYNNPADLFKVKNGILLVGGTGSYADLQFKGSA
LNFNTFQYDSGVNHSRTYIVWPYGRVAKRLNDKTVVAVDLTEPFNSNLDWGNDAFTRYAA
TQNYLTDVDLSPKISYAISKKLQIGGGINFNVLLKNEVNWAFPTGQSTYANLINRSSSFG
VGYNLGINYAVNDTNFLGITYYSRIRQNTSGTSYLGLAANPDFQFGFYMPATTVASYVHI
FNPKWLINLQVFQSEWNANQKVRLYNTAAPPPFTNFIFDMHFDASYAYLAAIRKQVSDKL
GIALAGMIDDGPEEDGLRTIVFPSDTQYFLGLIGDYRFTEHASMELILGHVYSNPSIQNK
AKVNNVPVPFTTGRVTINANVLDLKVKIEG

>DA38626 OmpP1/FadL, clinical isolate
MRLIFTGVLCLLTLNAQANVIQYFAGISYNNPADLFKVKNGILLVGGTGSYADLQFKGSA
LNFNTFQYDSGVNHSRTYIVWPYGRVAKRLNDKTVVAVDLTEPFNSNLDWGNDAFTRYAA
TQNYLTDVDLSPKISYAISKKLQIGGGINFNVLLKNEVNWAFPTGQSTYANLINRSSSFG
VGYNLGINYAVNDTNFLGITYYSRIRQNTSGTSYLGLAANPDFQFGFYMPATTVASYVHI
FNPKWLINLQVFQSEWNANQKVRLYNTAAPPPFTNFIFDMHFDASYAYLAAIRKQVSDKL
GIALAGMIDDDPEEDGLRTIVFPSDTQYFLGLIGDYRFTEHASMELILGHVYSNPSIQNK
AKVNNVPVPFTTGRVTINANVLDLKVKIEG

>BrisbaneLP07 OmpP1/FadL, environmental isolate
MRLIFTGVLCLLTLNAQANVIQYFAGISYNNPADLFKVKNGILLIGGTGSYADLQFKGSA
LNFNTFQYESGVNHSRTYIVWPYGRVAKRLNDKTVVAVDLTEPFNSNLDWGNDAFTRYAA
TQNYLTDVDLSPKISYAISKKLQIGGGINFNVLLKNEVNWAFPTGQSTYANLINRSSSFG
VGYNLGINYAVNDTNFLGITYYSRIRQNTSGTSYLGLAANPDFQFGFYMPATTVASYVHI
FNPKWLINLQVFQSEWNANQKVRLYNTAAPPPFTNFIFDMHFDASYAYLAAIRKQVSDKL
GIALAGMIDDGPEEDGLRTIVFPSDTQYFLGLIGDYRFTEHASMELILGHVYSNPSIQNK
AKVNNVPVPFTTGRVTINANVLDLKVKIEG

>BrisbaneLP47 OmpP1/FadL, clinical isolate
MLIGGTGSYADLQFKGSALNFNTFQ

>PHParis2001In2 OmpP1/FadL, environmental isolate
MRLIFTGVLCLLTLNAQANVIQYFAGISYNNPADLFKVKNGILLIGGTGSYADLQFKGSA
LNFNTFQYESGVNHSRTYIVWPYGRVAKRLNDKTVVAVDLTEPFNSNLDWGNDAFTRYAA
TQNYLTDVDLSPKISYAISKKLQIGGGINFNVLLKNEVNWAFPTGQSTYANLINRSSSFG
VGYNLGINYAVNDTNFLGITYYSRIRQNTSGTSYLGLAANPDFQFGFYMPATTVASYVHI
FNPKWLINLQVFQSEWNANQKVRLYNTAAPPPFTNFIFDMHFDASYAYLAAIRKQVSDKL
GIALAGMIDDGPEEDGLRTIVFPSDTQYFLGLIGDYRFTEHASMELILGHVYSNPSIQNK
AKVNNVPVPFTTGRVTINANVLDLKVKIEG

>PHHL01023034 OmpP1/FadL, clinical isolate
MRLIFTGVLCLLTLNAQANVIQYFAGISYNNPADLFKVKNGILLIGGTGSYADLQFKGSA
LNFNTFQYESGVNHSRTYIVWPYGRVAKRLNDKTVVAVDLTEPFNSNLDWGNDAFTRYAA
TQNYLTDVDLSPKISYAISKKLQIGGGINFNVLLKNEVNWAFPTGQSTYANLINRSSSFG
VGYNLGINYAVNDTNFLGITYYSRIRQNTSGTSYLGLAANPDFQFGFYMPATTVASYVHI
FNPK
